# Insight into Organization of Gliadin and Glutenin Extracted from Gluten Modified by Phenolic Acids

**DOI:** 10.3390/molecules28237790

**Published:** 2023-11-27

**Authors:** Renata Welc-Stanowska, Konrad Kłosok, Agnieszka Nawrocka

**Affiliations:** Institute of Agrophysics, Polish Academy of Sciences, Doświadczalna 4, 20-290 Lublin, Poland; k.klosok@ipan.lublin.pl (K.K.); a.nawrocka@ipan.lublin.pl (A.N.)

**Keywords:** Fourier transform infrared spectroscopy, gliadin, glutenin, secondary structure, phenolic acids

## Abstract

The changes in the secondary structure of individual gluten protein fractions (gliadin and glutenin) caused by the supplementation of model dough with eight phenolic acids were analysed. Gliadins and glutenins were extracted from gluten samples obtained from overmixed dough. The changes in the gliadin secondary structure depended on the amount of phenolic acid added to the dough. Higher acid concentrations (0.1% and 0.2%) led to a significant reduction in the amount of α-helices and to the formation of aggregates, non-ordered secondary structures, and antiparallel β-sheets. After the addition of acids at a lower concentration (0.05%), the disaggregation of pseudo-β-sheet structures and the formation of β-turns, hydrogen-bonded β-turns, and antiparallel β-sheets were detected. In the case of glutenin, most of the phenolic acids induced the formation of intermolecular hydrogen bonds between the polypeptide chains, leading to glutenin aggregation. When phenolic acids were added at a concentration of 0.05%, the process of protein folding and regular secondary structure formation was also observed. In this system, antiparallel β-sheets and β-turns were created at the expense of pseudo-β-sheets.

## 1. Introduction

Gluten proteins, which consist of gliadin and glutenin, make up one of the most complex protein aggregates in nature. Gliadins are soluble in aqueous alcohols but insoluble in water and salt solutions. Most gliadins are present as protein monomers with similar amino acid sequences. Based on electrophoretic mobility, gliadins are classified as α-/β-, γ- and ω-gliadins, with molecular weights ranging from 30 to 88 kDa [1]. In terms of secondary structure, α-/β- and γ-gliadins contain mainly α-helices and β-sheets, whereas ω-gliadins are dominated by β-turns [2]. Glutenins are polymeric proteins which are insoluble in alcohol solutions, with molecular weights ranging from 500 kDa to 10 MDa [3]. The glutenin fraction accounts for about half of the total gluten protein and is composed of macropolymers containing high-molecular-weight (HMW-GS) and low-molecular-weight glutenin subunits (LMW-GS). Both subunits are attached to each other via disulphide bonds [4]. Gliadins interact with glutenins through hydrogen bonding and non-covalent hydrophobic interactions. During wheat dough mixing, hydrated gliadins and glutenins form a three-dimensional gluten network, which is stabilized by S–S and non-covalent bonds [5]. One of the unique features of gluten proteins is that they contain exceptionally high amounts of glutamine (26–53%) and proline (10–29%), which are included in all epitopes and thus make them strong food allergens [6]. It is reported that gluten allergenicity can be reduced by biochemical modification of these proteins with polyphenols [7]. Waga suggested that β-turns structure are considered as the secondary structure responsible for allergenicity [8].

Plant polyphenols (phenolic acids, flavonoids, anthocyanins, etc.) are natural antioxidants occurring in fruits, cereals, herbs, or vegetables. They are responsible for free radical scavenging and prevent cardiovascular diseases, cancer, and diabetes and have a positive effect on eyesight and brain functions [9,10]. Polyphenols interact with proteins rich in proline, such as gliadin and glutenin. Proline residues are considered as potential binding sites for these bioactive compounds. Polyphenols can interact with proteins reversibly (e.g., hydrogen bonding and van der Waals forces) and irreversibly (covalent bonds) [11,12].

The supplementation of wheat dough with some bioactive compounds seems to be an efficient method to introduce pro-health components into wheat bread. On the other hand, the addition of different bioactive compounds (e.g., dietary fibre, polyphenols, and anthocyanins) results in bread quality reduction due to the disruption of the gluten network. The interactions between gluten and different chemical compounds have been studied by many research groups. It was reported that the addition of dietary fibre preparations to model dough caused the formation of new hydrogen bonds and changes in the conformation of the disulphide bridges, leading to gluten protein aggregation [13]. The addition of phenolic acids also resulted in undesirable changes in the secondary structure of the gluten network [14,15,16]. Sivam et al. (2012) studied the interaction between gluten proteins and pectins, as well as polyphenols extracted from berries, in a model and in real bread. The authors observed a decrease in the amount of β-sheets at the expense of β-turns and a greater fraction of unordered structures in both breads [17]. The interactions between gliadin protein and anthocyanins extracted from wheat dough were also studied. The results showed that anthocyanins caused a decrease in the amount of unordered structures with the simultaneous formation of new hydrogen bonds. Additionally, a significant decrease in the amount of β-turns and the content of the most stable disulphide bridges was observed. This indicated abnormal protein folding or its aggregation [7,18]. 

In our previous studies, we analysed the interactions between gluten (as a mixture of gliadin and glutenin) and two groups of phenolic acids that differed by the presence of one additional double bond between the carboxyl group and the aromatic ring [16,19]. We suggested that the mechanism of gluten–phenolic acid interactions depends on the size of the phenolic acid and/or the type of functional groups on the aromatic ring of the acid. The aim of our studies was to determine the effect of selected phenolic acids on the secondary structure and functionality of individual gluten protein fractions (gliadins and glutenins) in the dough during the overmixing process. The gliadin and glutenin fractions were extracted from the model dough supplemented with phenolic acids, and the changes in the secondary structure of the individual fractions were analysed with the application of FTIR spectroscopy. Two groups of phenolic acids were analysed: hydroxycinnamic acid derivatives (caffeic, ferulic, coumaric, and sinapic acid) and hydroxybenzoic acid derivatives (protocatechuic, vanillic, 4-hydroxybenzoic, and syringic acid).

## 2. Results

### 2.1. Changes in the Secondary Structure of Gliadin 

The amide I band (1570–1720 cm^−1^), which is mainly connected with the C=O stretching vibrations, is particularly sensitive to the secondary structure of proteins due to the characteristic hydrogen bond pattern between the amide C=O and N-H groups. The secondary structure of proteins can also be determined based on amide III band analysis. The amide III region provides information which is complementary to the amide I band, and the analysis of the protein’s secondary structure is not disturbed by water oscillations [20]. In order to determine the structural changes within individual gluten proteins (gliadins and glutenins) as a result of phenolic acid supplementation, the FTIR spectra were baseline corrected and field normalized in the amide I and amide III regions. In the next step, the difference spectra were calculated by the subtraction of the control sample spectrum from the spectra of the modified samples.

**Figure 1:**
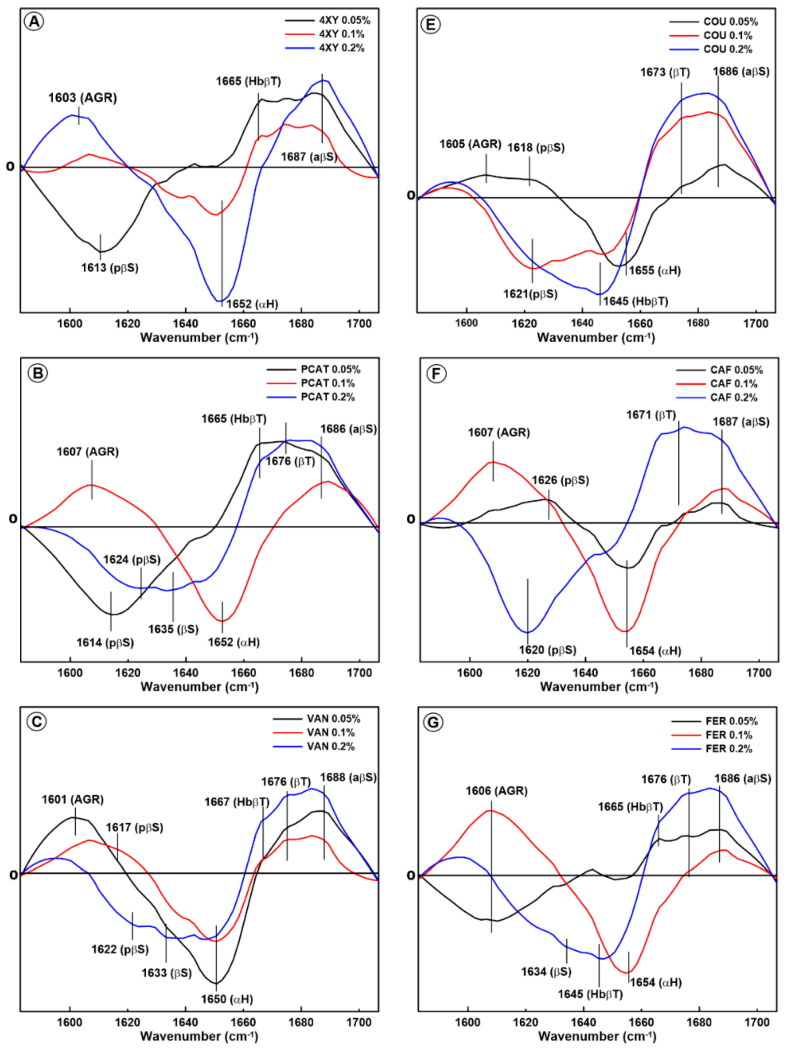

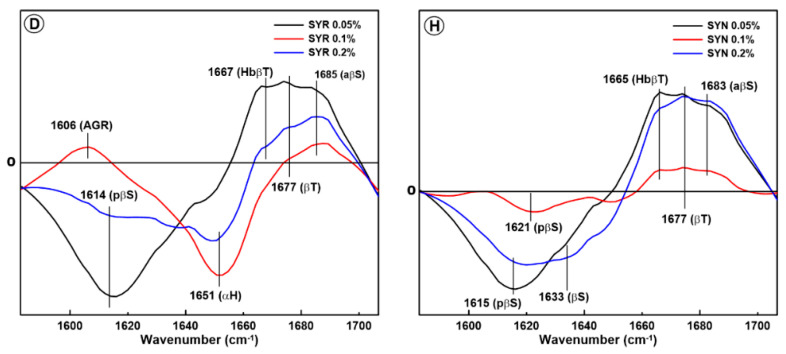
Difference spectra in the amide I band showing changes in the secondary structure of gliadin extracted from the model dough supplemented with eight phenolic acids with content levels of 0.05%, 0.1%, and 0.2%: 4-xydroxybenzoic acid (4-XY) (**A**), coumaric acid (COU) (**E**), protocatechuic acid (PCAT) (**B**), caffeic acid (CAF) (**F**), vanillic acid (VAN) (**C**), ferulic acid (FER) (**G**), syringic acid (SYR) (**D**), and sinapic acid (SYN) (**H**). The following secondary structures are marked: AGR-aggregates, HbβT-hydrogen-bonded β-turns, αH-α-helix, βT-β-turns, pβS-pseudo-β-sheets, aβS-antiparallel β-sheets, and βS-β-sheet structures.

The difference spectra in the amide I band obtained for the gliadin proteins are presented in Figure 1. As can be seen, the addition of phenolic acids to the model dough induced changes in the spectral regions; assigned to the following secondary structures present in proteins: aggregates (ca. 1600 cm^−1^); β-sheets (ca. 1630 cm^−1^); α-helices (ca. 1650 cm^−1^); β-turns (ca. 1640 and 1670 cm^−1^); hydrogen-bonded β-turns (ca. 1665 cm^−1^ and ca. 1645 cm^−1^); pseudo-β-sheets (ca. 1620 cm^−1^); and antiparallel β-sheets (ca. 1680 cm^−1^) [13,21,22,23,24,25]. The term “pseudo-β-sheets” is adopted by us and is assigned to hydrogen-bonded β-sheets that have taken the form of antiparallel β-sheets [13]. 

Hydrogen-bonded β-turns can be detected in two spectral regions because two kinds of hydrogen bonds can be formed within them: intra- (ca. 1645 cm^−1^) and interchain (ca. 1665 cm^−1^). If the carbonyl groups in the β-turns are not hydrogen-bonded, the band at 1670 cm^−1^ is observed. If the carbonyl groups become hydrogen-bonded, a shift of their absorption band toward lower wavenumbers should be expected; therefore, the band at 1665 cm^−1^ can be assigned to β-turns with interchain hydrogen bonds [25]. The appearance of the band at 1642 cm^−1^ can indicate the possibility of the formation of internal hydrogen bonds, especially intramolecular bonds [22]. The band at ca. 1640–1650 cm^−1^ can also be assigned to the random coils [26]. However, we decided that this band was probably connected with the hydrogen-bonded beta-turns because we also observed a band connected with non-hydrogen-bonded beta-turns at ca. 1670 cm^−1^. In all cases, the orientations of these two bands were opposite. For this reason, we assigned the band at ca. 1643–1645 cm^−1^ to the hydrogen-bonded beta-turns rather than to random coils. In most of the samples, three characteristic spectral regions were detected: negative bands associated with α-helices and/or β-sheets and positive bands connected with β-turns, hydrogen-bonded β-turns, antiparallel β-sheets, and aggregates. The intensity of these bands was not unambiguously related to the acid concentration.

The supplementation of the model dough with phenolic acids belonging to the hydroxycinnamic group as well as the hydroxybenzoic group induced changes in the gliadin secondary structures which can be divided into two groups. It was not possible to link the structural changes clearly to a specific group of acids; however, we noticed two trends. In the first group of changes, the reduction in the amount of α-helices with the simultaneous increase in the content of aggregates, antiparallel β-sheets, β-turns, and hydrogen-bonded β-turns was observed. This effect was detected for the samples modified with hydroxybenzoic as well as hydroxycinnamic acids (0.1% 4XY, 0.2% 4XY, 0.1% PCAT, 0.05–0.2% VAN, 0.1% SYR, 0.005% COU, 0.1%, 0.1% CAF, and 0.1% FER). The significant reduction in the content of α-helices suggests that the aggregates, antiparallel β-sheets, β-turns, and hydrogen-bonded β-turns observed after the phenolic acid addition are formed at the expense of α-helices. As hydrogen-bonded β-turns belong to aggregated structures, their presence, as well as the presence of aggregates, suggests that the gliadins lose their native basic secondary structure after the addition of phenolic acids to the model dough. Additionally, the greater amount of β-turns indicates that gliadin proteins adopt non-regular secondary structures. The reduction in the content of α-helices and the destruction of the ordered structure of gliadin protein was also observed by Feng et al. as a result of an interaction between gliadin and wheat bran [27]. It is also reported that the presence of bands in the spectral region (1611–1630) cm^−1^ can be connected with the fibrillation of native globular proteins rich in β-sheets and amyloid formation [28]. Despite the fact that the dominant structure of gliadin is α-helix, these proteins are also rich in β-sheet structures [29]. The formation of an amyloid structure within gliadin polypeptide chains induced by selected phenolic acids was suggested in our previous studies [30], where phenolic acids were added to gliadin ”from outside”, and the model complexes were formed.

The second group of changes In the gliadin secondary structure Induced by the phenolic acids (hydroxybenzoic as well as hydroxycinnamic) are connected with the formation of hydrogen-bonded β-turns and antiparallel β-sheets at the expense of pseudo-β-sheet (pβS) structures. This effect was detected for 0.05% 4XY, 0.05 and 0.2% PCAT, 0.05 and 0.2% SYR, 0.1% COU, 0.05% FER, and 0.05% and 0.2% SYN. Mangavel et al. reported that the presence of the band associated with pβS is connected with the aggregation of gliadin proteins [31]. Therefore, the negative band assigned to pseudo-β-sheets indicates that the addition of phenolic acids results in the disaggregation of these β-structures and probably leads to hydrogen bonding between β-turns. According to Juszczyk et al., pseudo-β-sheets are stabilized by strong, intermolecular hydrogen bonds [32]. This suggests that the modification of model dough with phenolic acids results in the cleavage of the intermolecular bonds within the pseudo-β-sheets in gliadin proteins. On the other hand, Kłosok et al. reported that an increase in the content of antiparallel β-sheets and β-turns at the expense of pseudo-β-sheets can be caused by prolonged dough mixing [14]. A similar effect was observed by Krekora et al., who analysed the interactions between gluten and phenolic acids in the samples taken after dough breakdown [33]. According to Lancelot et al. (2021), a greater amount of antiparallel β-sheets indicates protein folding and the formation of more ordered secondary structures [34]. Interestingly, this kind of change in the gliadin secondary structure is observed in most of the samples with the lowest phenolic acid concentration (0.05%).

**Figure 2:**
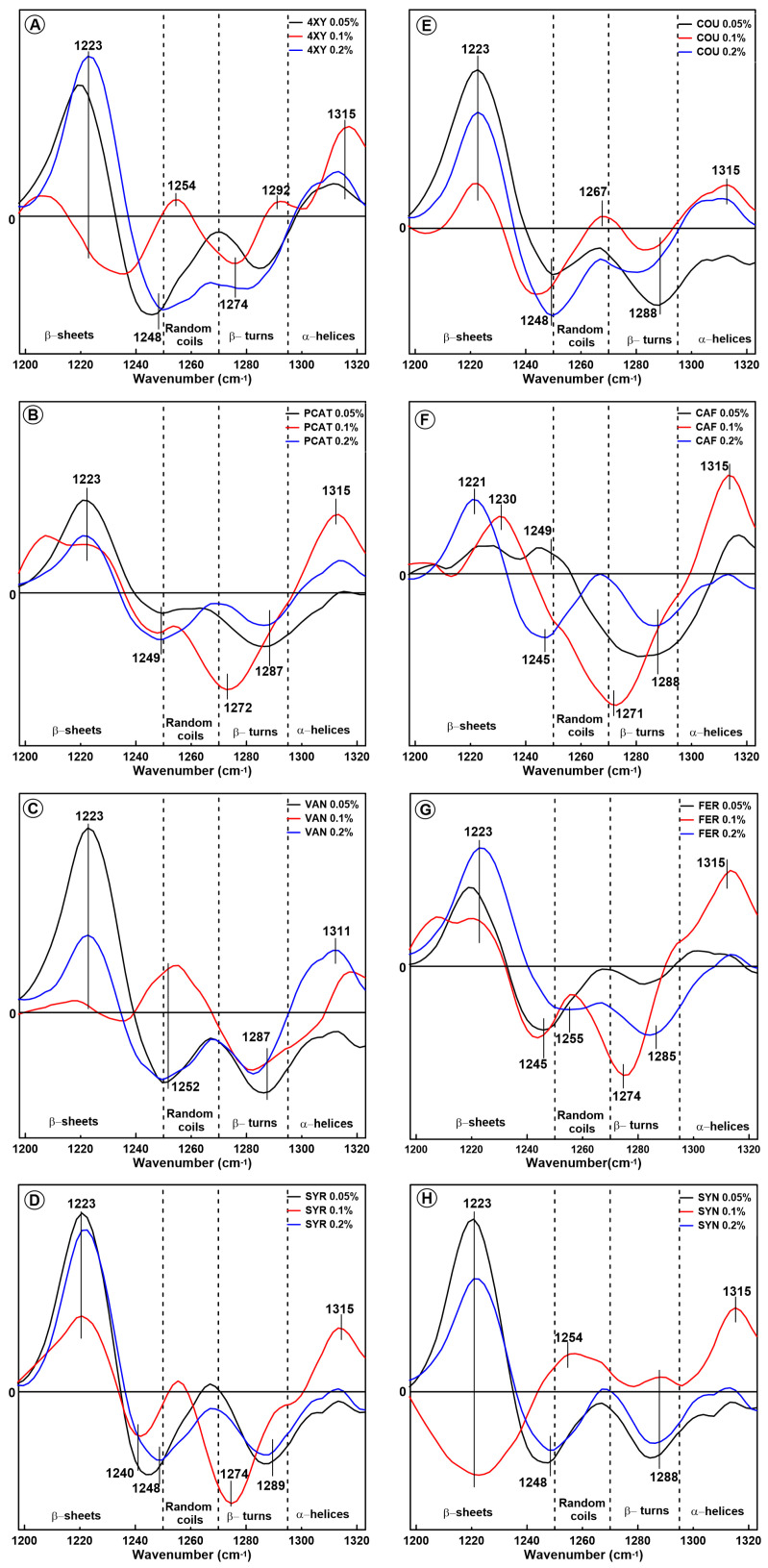
Difference spectra in the amide III band obtained for gliadin extracted from the model dough supplemented with eight phenolic acids with content levels of 0.05%, 0.1%, and 0.2%: 4-xydroxybenzoic acid (4-XY) (**A**), coumaric acid (COU) (**E**), protocatechuic acid (PCAT) (**B**), caffeic acid (CAF) (**F**), vanillic acid (VAN) (**C**), ferulic acid (FER) (**G**), syringic acid (SYR) (**D**), and sinapic acid (SYN) (**H**).

In order to obtain additional information about changes in the gliadin secondary structure, the amide III region was also analysed. The difference spectra in the amide III band for the gliadin extracted from the model dough supplemented with phenolic acids are presented in Figure 2. In general, the region 1200–1340 cm^−1^ (amide III band) can be divided into four spectral ranges assigned to β-sheets (1200–1250 cm^−1^), random coils (1250–1270 cm^−1^), β-turns (1270–1295 cm^−1^), and α- helices (1295–1330 cm^−1^) [35]. Analysis of the spectra showed that the gliadin secondary structures after interaction with phenolic acids contain more β-sheets compared to the control sample (ca. 1223 cm^−1^). Additionally, in most cases, positive bands which differ in intensity are observed in the region assigned to α-helices (ca. 1315 cm^−1^). The intensity of these bands was the highest when phenolic acids were added at a concentration of 0.1% (except for VAN). This result may seem inconsistent with the data obtained from the amide I analysis. However, the amide III band provided more general information about the secondary structure of proteins in comparison with the amide I band. For this reason, the amide I band is regarded as a basic band when used in the analysis of the secondary structure of proteins. Therefore, the band at 1315 cm^−1^ observed in the amide III region probably did not originate only from α-helices. This band can originate partially from α-helices despite the fact that the amide I band shows a negative band connected with this secondary structure. This band can be also connected with the presence of phenolic acids in the sample. The spectra of pure phenolic acid in the amide III band, which are presented in Figure S1 in the Supplementary Material, showed a band at a similar wavenumber. The relationship between the band intensity and the acid concentration was not observed, and it was not taken into account during the analysis of the difference spectra in the amide III region. However, the interactions between the gliadins and phenolic acids can affect the intensity of the chemical bonds’ oscillations in the phenolic acid molecule. For this reason, we can observe a positive band in the spectral region that is characteristic for α-helices for samples modified by 4-hydroxybenzoic, coumaric, and syringic acids because these acids have bands at ca. 1310 cm^−1^ (see Figure S1 in the Supplementary Material). 

In the case of gliadins extracted from the dough modified by FER, SYR, PCAT, CAF, and 4XY, a significant reduction in the amount of β-turns can be seen. Wellner et al. [36], who analysed structural changes in the gluten network during mixing, reported that the amount of random coils decreased over time, whereas the amount of β-turns and β-sheets increased over time as the gluten dough was mixed. The authors suggested that the increase in β-sheet structures was associated with the hydration of high-molecular-weight glutenin subunits; therefore, this effect should not be observed in the case of gliadin proteins. On the other hand, the presence of the strong band at 1223 cm^−1^ in the gliadin samples can be explained based on the studies of [37]. According to the authors, the amide III band provides important information about the types of hydrogen bonds which are formed between β-sheets in the gluten network [37]. The authors distinguished two types of hydrogen bonds and suggested that the type I H-bonds (-NH⋯O=C-) and the type II (-NH⋯O) can be associated with characteristic bands at ca. 1235 cm^−1^ and 1225 cm^−1^, respectively. According to Nawrocka et al., the type I H-bonds can be formed between gluten polypeptide chains as intermolecular bonds, and this process leads to protein aggregation [38]. The band at 1235 cm^−1^ is not observed in the amide III difference spectra obtained for the gliadin proteins. The hydrogen bond -NH⋯O, which is associated with the intensive band at 1223 cm^−1^, can be formed between the polypeptide chain and hydroxyl group attached to the aromatic ring of phenolic acids. The presence of these bonds indicates that phenolic acids are incorporated between the gliadin polypeptide chains. Additionally, in the case of 0.05% SYR, PCAT, and SYN, the greatest intensity of the band at 1223 cm^−1^ in the amide III band correlates with the greatest reduction in the content of pseudo-β-sheets in the amide I range. This effect indicates that the addition of these three acids at the lowest concentration resulted in pseudo-β-sheet disaggregation and the formation of ordered secondary structures. This effect indicates that the addition of phenolic acids at a low concentration (0.05%) results in pseudo-β-sheet disaggregation and ordered secondary structure formation. It suggests that in this case the phenolic acids are located between the gliadin polypeptide chains and cause chain separation. It may seem that the incorporation of phenolic acids within the gliadin structure should be manifested by the presence of the bands which are characteristic for pure phenolic acids. However, the content of acids compared to gluten and starch is very low (0.05–0.2%), and the band characteristics for phenolic acids cannot be clearly visible. The spectra of pure phenolic acids are presented in Figure S1. It can be concluded that the presence of a strong band at 1223 cm^−1^ can be connected with both the formation of β-sheets from pseudo-β-sheets and the interaction between phenolic acids and gliadin polypeptides via hydrogen bonds. It can be also seen that the intensity of the band at 1223 cm^−1^ is correlated with the intensity of the band associated with α-helices (1315 cm^−1^). The greater the amount of α-helices, the lower the observed intensity of the band at 1223 cm^−1^. 

In general, the structural changes within gliadin proteins cannot be clearly assigned to a specific group of phenolic acids. Hydroxybenzoic acids as well as hydroxycinnamic acids have similar functional groups attached to the aromatic ring, and the observed structural changes are common for both groups. However, we noticed that the changes in the secondary structure of the gliadin proteins depends on the amount of phenolic acid added to the model dough. The presence of some phenolic acids at higher concentrations (0.1% XY, 0.2% XY, 0.1% PCAT, 0.1% CAF, and 0.1% FER) caused a significant reduction in the amount of α-helices, with the simultaneous formation of aggregates, ordered aggregates, and antiparallel β-sheets. The addition of SYR, SYN, and PCAT at a concentration of 0.05% resulted in the disaggregation of the pseudo-β-sheet structures and the formation of β-turns and hydrogen-bonded β-turns. It is likely that the presence of phenolic acids at a low concentration results in their location between gliadin polypeptide chains and polypeptide separation, but this amount is not enough to disturb the whole protein structure during dough mixing. It can be concluded that the bonds within the gliadin structure, which are broken during dough mixing, can be formed again in the presence of a small amount of phenolic acids. When the concentration of acids is higher, the formation of hydrogen bonds between the gliadin polypeptide chains can be blocked by a molecule of phenolic acid; therefore, the protein structure is destroyed, and a greater amount of aggregates and non-regular structures is observed.

### 2.2. Changes in the Secondary Structure of Glutenin

**Figure 3:**
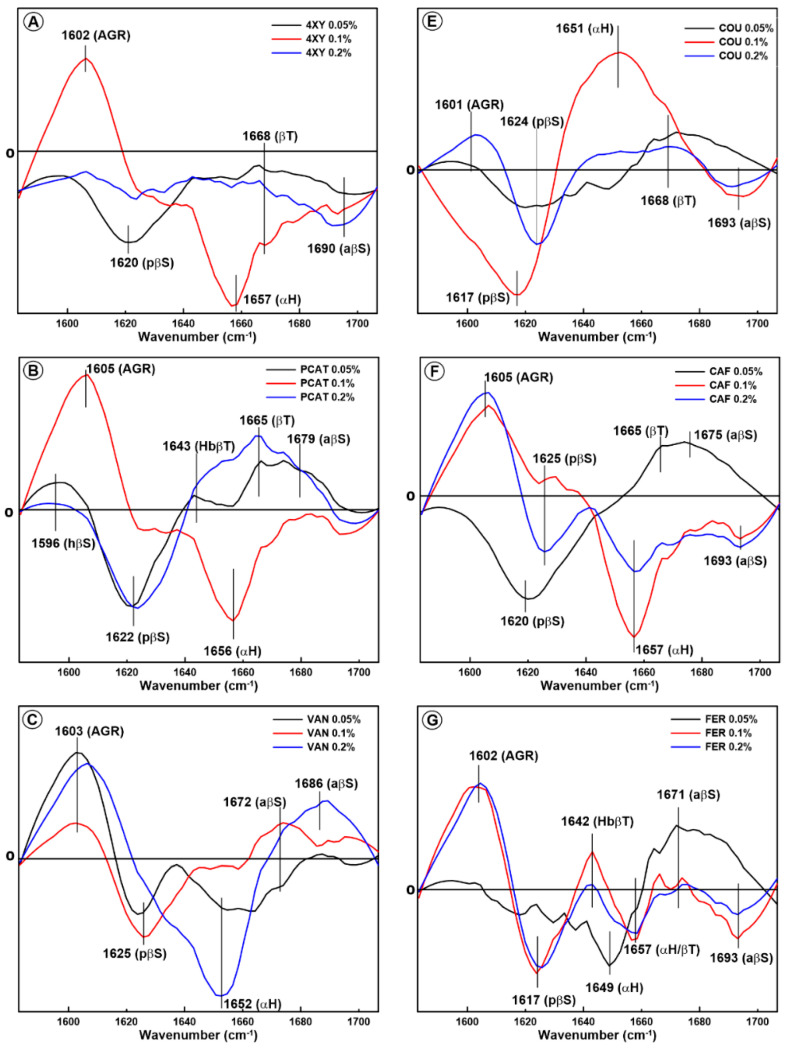

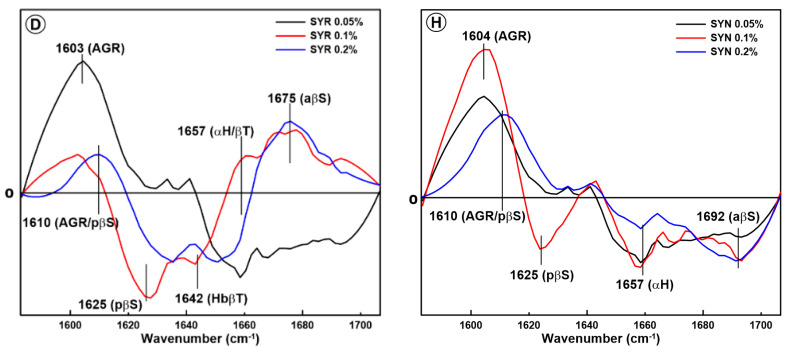
Difference spectra in the amide I band showing changes in the secondary structure of glutenin obtained from the model dough supplemented with eight phenolic acids with content levels of 0.05%, 0.1%, and 0.2%: 4-xydroxybenzoic acid (4-XY) (**A**), coumaric acid (COU) (**E**), protocatechuic acid (PCAT) (**B**), caffeic acid (CAF) (**F**), vanillic acid (VAN) (**C**), ferulic acid (FER) (**G**), syringic acid (SYR) (**D**), and sinapic acid (SYN) (**H**). The following secondary structures are marked: AGR-aggregates, HbβT-hydrogen-bonded β-turns, αH-α-helix, βT-β-turns, pβS-pseudo-β-sheets, aβS-antiparallel β-sheets, and βS-β-sheet structures.

The changes in the secondary structure of glutenin extracted from model dough supplemented with phenolic acids were also determined based on the difference spectra calculated in the amide I and amide III regions. The difference spectra in the amide I region obtained by subtraction of the spectrum of the control sample (unmodified glutenin proteins) from the spectra of the samples modified by phenolic acids are presented in Figure 3. 

As can be seen, the interaction between the glutenin and phenolic acids during dough mixing induces changes in the glutenin secondary structure; these changes can be divided into three groups. In the first case, a significant increase in the amount of protein aggregates (ca. 1600 cm^−1^), with the simultaneous presence of a strong negative band assigned to the α-helices (1650 cm^−1^), is observed. It is well known that glutenin fractions comprise protein aggregates; however, the addition of selected phenolic acids to model dough induced even stronger glutenin aggregation. This effect was observed for 0.1% XY, 0.1% PCAT, 0.2% VAN, 0.05% SYR, 0.1% CAF, and 0.2% CAF. It can be seen that glutenin aggregates are formed primarily at the expense of α-helices. In the case of 0.2% VAN, in addition to aggregates, antiparallel β-sheets structures are formed when the amount of α-helices decreases. 

In the second case, a negative band associated with pseudo-β-sheets (ca. 1620 cm^−1^), with the simultaneous presence of positive bands connected with β-turns and/or antiparallel β-sheets, is observed. This effect is detected in the case of the supplementation of model dough with PCAT (0.05% and 0.2%), VAN (0.1%), SYR (0.1% and 0.2%), and CAF (0.05%). A similar effect was observed in the gliadin protein extracted from the dough supplemented with the lowest phenolic acid concentration (0.05%).

In the case of the FER and SYN acids, except for FER 0.05%, the strongest changes are visible in the spectral region associated with glutenin aggregates. Additionally, a slight reduction in the amount of pseudo-β-sheets, β-turns, α-helices, and antiparallel β-sheets is observed in these samples. 

Completely different changes in the secondary structure of glutenin, compared to other phenolic acids, are detected as a result of the interaction between protein and COU at a concentration of 0.1%. For this sample, a significant reduction in the amount of pseudo-β-sheets, with a simultaneous significant increase in the content of α-helices, is observed. It can be concluded that in this system, the coumaric acid addition resulted in a disaggregation of glutenin polypeptide chains and an ordered secondary structure formation. 

The amide I band analysis showed that phenolic acids interact with glutenin in a different way than they do with gliadins. The changes in the glutenin secondary structure are more heterogenous and cannot be divided into two groups as in the case of gliadins. On the other hand, it seems that the addition of most of the phenolic acids belonging to the hydroxybenzoic group resulted in the strongest reduction in the amount of α-helices. 

**Figure 4:**
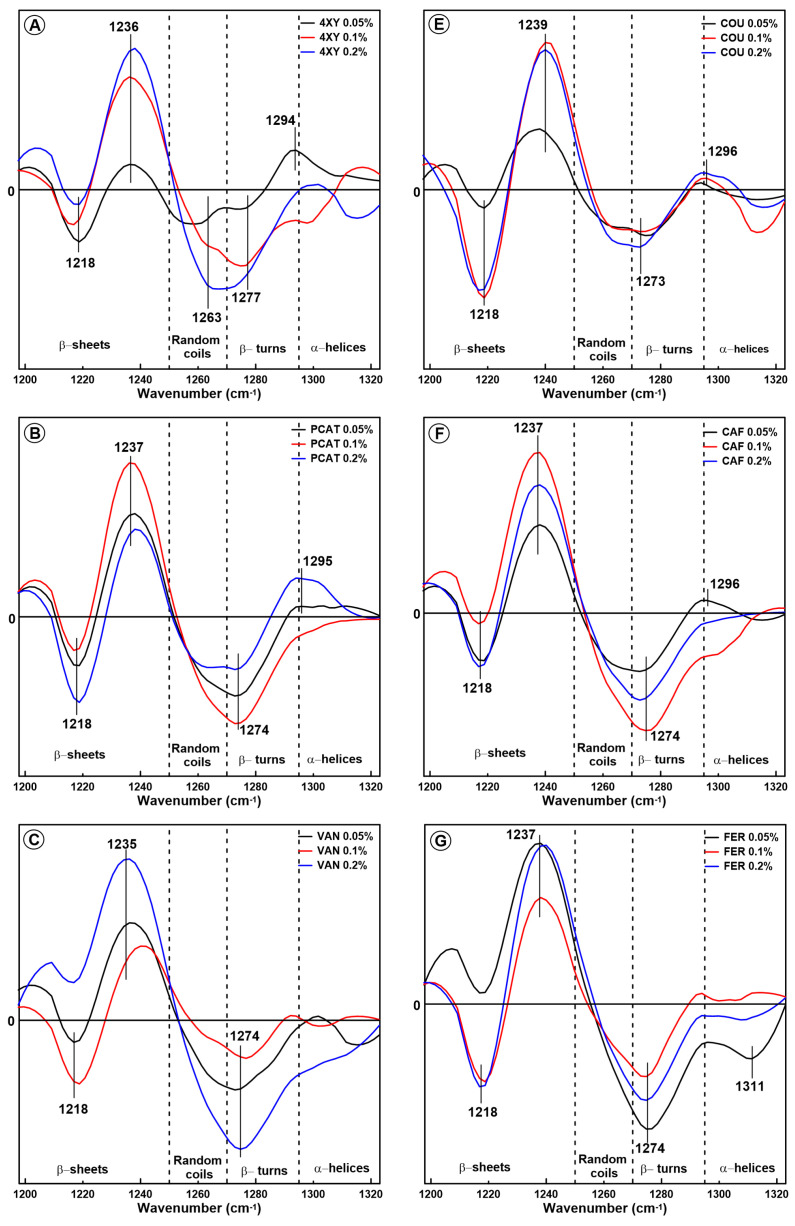

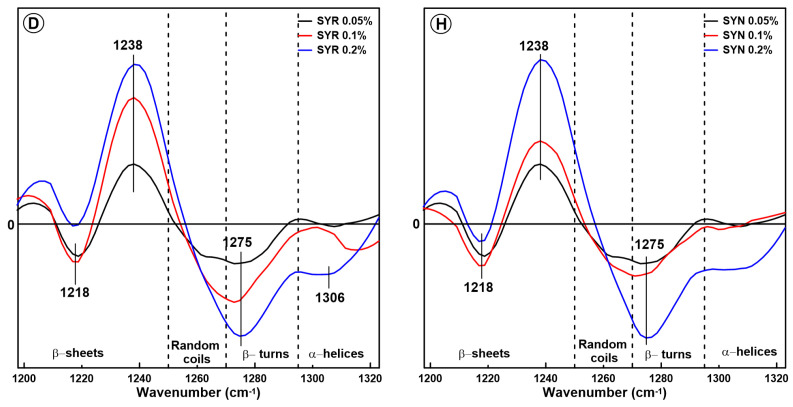
Difference spectra in the amide III band obtained for glutenin extracted from the model dough supplemented with eight phenolic acids with content levels of 0.05%, 0.1%, and 0.2%: 4-xydroxybenzoic acid (4-XY) (**A**), coumaric acid (COU) (**E**), protocatechuic acid (PCAT) (**B**), caffeic acid (CAF) (**F**), vanillic acid (VAN) (**C**), ferulic acid (FER) (**G**), syringic acid (SYR) (**D**), and sinapic acid (SYN) (**H**).

The difference spectra in the amide III band obtained for glutenin are presented in Figure 4. As can be seen, in the case of model dough supplemented with hydroxybenzoic as well as hydroxycinnamic acids, all the spectra calculated for glutenin contain negative and positive bands in the spectral range associated with β-sheets (ca. 1218 cm^−1^ and 1236 cm^−1^, respectively) and negative bands in the range connected with β-turns (ca. 1274 cm^−1^). It is reported that the β-sheet structure is the main secondary structure in glutenin proteins. Moreover, β-sheets are considered to be the most stable protein conformation [39]. A reduction in the amount of β-sheets for glutenin proteins and a decrease in protein stability was observed by Feng et al. as a result of glutenin interaction with dietary fibre and ferulic acid [27]. In other studies, Feng et al. showed that the dough mixing leads to the formation of a greater amount of β-sheets in the glutenin macropolymer [40]. Wellner et al. (2005) [36] claimed that the amount of β-sheets increased over time as the gluten dough was mixed and that this effect was associated with the hydration of high-molecular-weight glutenin subunits.

On the other hand, as mentioned above, the band at ca. 1235 cm^−1^ can be also associated with the presence of the type I H-bonds (-NH⋯O=C-). Interestingly, in the case of gliadin protein, the presence of phenolic acids resulted in the formation of type II H-bonds (-NH⋯O). The bonds -NH⋯O=C- can be formed between protein polypeptide chains as intermolecular bonds, and this process leads to protein aggregation. Additionally, according to Anderle and Mendelsohn (1987), the band at ca. 1235 cm^−1^ can be connected with β-sheets as well as antiparallel β-sheets with inter- and intrachain hydrogen bonds [20]. The presence of this band is also regarded as a sign of protein aggregation. This result is in agreement with the data obtained from the amide I band analysis because in most of the samples an increase in the amount of antiparallel β-sheets is observed.

It can be concluded that changes in the secondary structure of glutenin induced by phenolic acids are more heterogenous than in the case of gliadin. This is probably connected with the fact that glutenin belongs to the largest and most complex proteins in nature; these proteins contain fractions that vary in size significantly [41]. Most of the phenolic acids induced the formation of intermolecular hydrogen bonds between the polypeptide chains, leading to glutenin aggregation. In the case of PCAT, FER, and CAF added at the lowest concentration (0.05%), the process of protein folding and regular secondary structure formation was observed, since antiparallel β-sheets and β-turns were formed at the expense of pseudo-β-sheets. A similar effect was observed for gliadin extracted from the model dough supplemented with SYR, SYN, and PCAT at a concentration of 0.05%. 

## 3. Materials and Methods

### 3.1. Materials

Wheat starch, wheat gluten, coumaric (COU), 4-hydroxybenzoic (4-XY), ferulic (FER), caffeic (CAF), vanillic (VAN), and protocatechuic acid (PCAT) were purchased from Sigma Aldrich (Poznań, Poland). Syringic acid (SYR) was purchased from Alfa Aesar (Gdańsk, Poland) and sinapic acid (SYN) was purchased from Apollo Scientific (Manchester, UK). Ethanol was obtained from Avantor (Gliwice, Poland). All reagents were of at least analytical grade. Double-distilled water was used.

### 3.2. Dough Preparation

The model dough samples supplemented with eight phenolic acids (4-XY, VAN, FER, CAF, COU, PCAT, SYR, SYN) were prepared in a farinograph-E (Brabender, Duisburg, Germany), according to the method described by Welc et al. [16]. Briefly, model flour was prepared from commercially available wheat starch and wheat gluten (at the same moisture basis) by mixing them at a constant weight ratio of 80:15 (*w*/*w*). The simplified composition of the model flour was used because of the absence of natural dietary fibre and polyphenols in the model flour, which could interact with the gluten network and cause some changes in its structure. The reconstituted flour can be regarded as a good representative of a common wheat flour because the weight proportion of commercially available wheat gluten and wheat starch in the reconstituted flour was assumed on the basis of the real contents of starch and gluten in a common wheat flour. This artificial model system facilitates the study of structural changes in gluten proteins as a result of phenolic acid addition. The phenolic acids were added in concentrations of 0.05%, 0.1%, and 0.2% in relation to the model flour–phenolic acid mixture weight. Water absorption was kept at a constant level of 65%. In order to ensure destruction of the gluten network in the model dough as a result of dough supplementation with phenolic acids, the mixing process was extended to 60 min for all samples. Dough samples were taken directly after the end of the mixing process.

### 3.3. Fourier Transform Infrared Spectra (FT-IR)

The FT-IR spectra were recorded with a Nicolet 6700 FT-IR spectrometer (Thermo Scientific, Waltham, MA, USA) equipped with a diamond ATR attachment. Spectra collection and data manipulation were carried out according to a procedure developed by Nawrocka et al. [38]. The samples were analysed in the powder form. The analysed spectra were averaged over five registered spectra. The ORIGIN (v.2019b PRO, OriginLab Corporation, Northampton, MA, USA) was used to process the recorded spectra (Nawrocka et al., 2018 [38]).

### 3.4. Gliadins and Glutenins Preparation

The gliadins were extracted from powdered gluten according to the method described by Taddei et al., with some modification [7]. Briefly, the sample was dissolved in 40 mL of 70% ethanol (120 mg) and stirred on a magnetic stirrer at room temperature (25 °C) for 4 h. In the next step, the mixture was centrifuged at 4000× *g* for 10 min. The supernatant containing gliadin proteins was then collected into a flask and lyophilized to obtain powdered gliadins for further analysis. To ensure complete extraction of the gliadins, the process was repeated three times. The ethanol-insoluble protein fractions obtained after centrifugation were the glutenin proteins.

## 4. Conclusions

Changes in the secondary structure of gliadin proteins extracted from model dough depend on the amount of phenolic acid added to the dough. The presence of some phenolic acids at higher concentrations (0.1% XY, 0.2% XY, 0.1% PCAT, 0.1% CAF, and 0.1% FER) caused a significant reduction in the amount of α-helices, with a simultaneous formation of aggregates, ordered aggregates, and antiparallel β-sheets. The addition of SYR, SYN, and PCAT at a concentration 0.05% resulted in the disaggregation of pseudo-β-sheet structures and the formation of β-turns and hydrogen-bonded β-turns. The presence of phenolic acids at a low concentration result in their location between the gliadin polypeptide chains and polypeptide separation. When the concentration of acids is higher, the formation of hydrogen bonds between the gliadin polypeptide chains is disturbed; therefore, the protein structure is destroyed and a greater amount of aggregates is observed. In the case of glutenin, changes in the protein secondary structure are more heterogenous than in the case of gliadin. Most of the phenolic acids induced the formation of intermolecular hydrogen bonds between the polypeptide chains, leading to glutenin aggregation. In the case of PCAT, FER, and CAF added at the lowest concentration (0.05%), the process of protein folding and regular secondary structure formation was observed, since antiparallel β-sheets and β-turns were formed at the expense of pseudo-β-sheets.

In the case of gliadin, as well as glutenin, changes in the secondary structure of proteins cannot be unambiguously connected with the chemical structure of phenolic acids. Phenolic acids belonging to the hydroxybenzoic and hydroxycinnamic groups induced heterogenous changes in the gliadin and glutenin organization.

## Data Availability

Data are contained within the article and supplementary materials.

## Supplementary Materials

The following supporting information can be downloaded at: https://www.mdpi.com/article/10.3390/molecules28237790/s1, Figure S1: The spectra of pure phenolic acids detected in amide III region.
