# Supplementary material for: Insight into Organization of Gliadin and Glutenin Extracted from Gluten Modified by Phenolic Acids
Source: Molecules. 2023 Nov 27;28(23):7790. doi: 10.3390/molecules28237790 (PMC10708489; doi:10.3390/molecules28237790)
Supplement: Supplementary file 1 [file molecules-28-07790-s001.zip › molecules-2638124-supplementary.pdf]

Supplementary Material

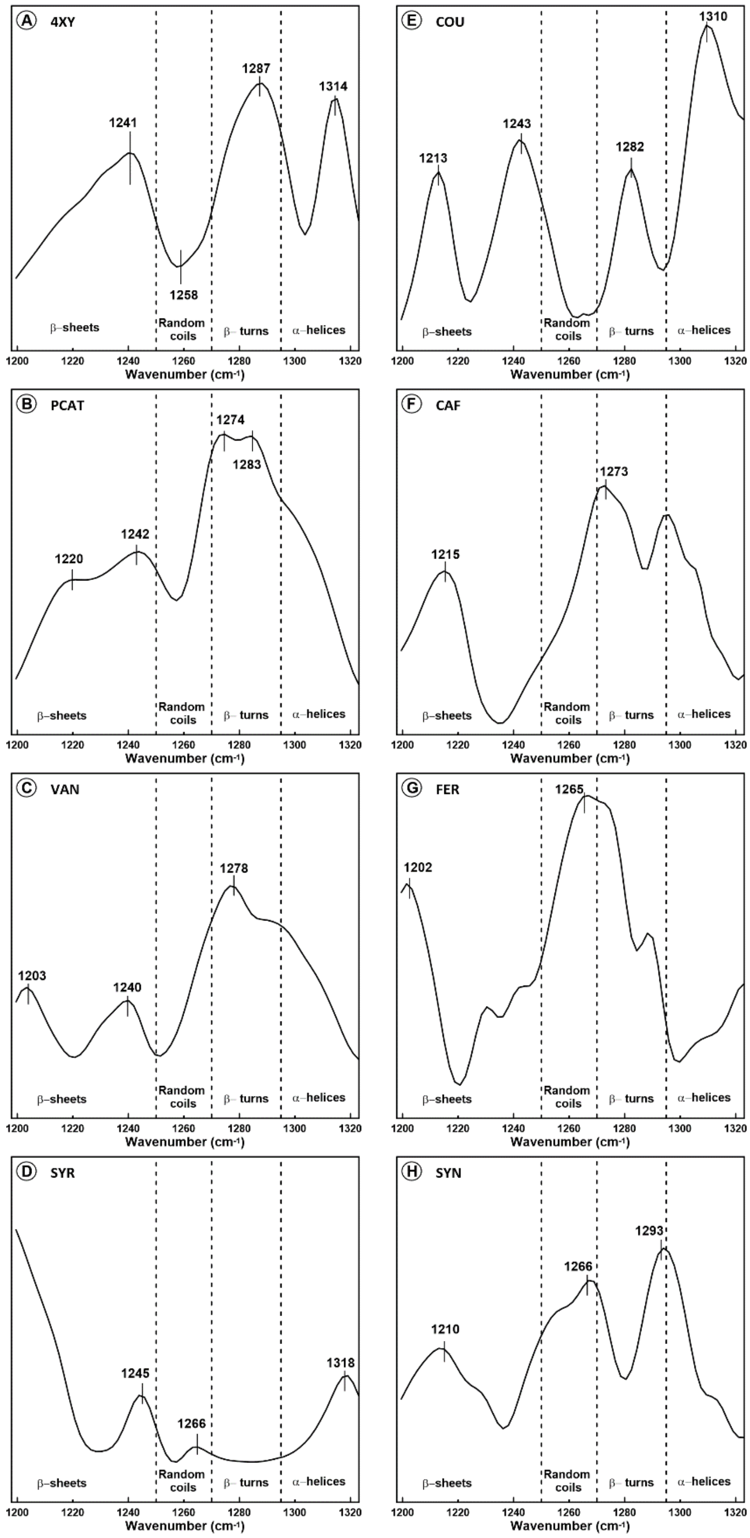

Figure S1 The spectra of pure phenolic acids detected in amide III region.
